# Changes in Food Waste among a Sample of U.S. Consumers after Beginning Anti-Obesity Medication

**DOI:** 10.3390/nu16193274

**Published:** 2024-09-27

**Authors:** Jamil Mansouri, Brian E. Roe

**Affiliations:** 1 Department of Agricultural Economics, Purdue University, West Lafayette, IN 47907, USA; mansouj@purdue.edu; 2 Department of Agricultural, Environmental and Development Economics, Ohio State University, Columbus, OH 43210, USA

**Keywords:** food waste, anti-obesity medications, glucagon-like peptide-1 agonist, U.S. consumers, survey

## Abstract

Background/Objectives: Glucagon-like peptide-1 agonists (GLP1As) are increasingly prescribed to treat obesity. While studies document how these medications impact dietary habits, their relationship to consumer food waste is unexplored. Approximately one-third of all food is wasted, which creates substantial economic and environmental damage. The purpose of this study is to assess how consumers alter food waste after beginning GLP1As and to identify factors associated with this relationship. Methods: Retrospectively reported changes in the amount of food wasted since beginning a GLP1A are gathered from a sample of 505 U.S. consumers via a self-administered online survey. Regression analysis yields associations between changes in post-GLP1A-uptake food waste and the length and type of medication use, medication side effects, post-uptake changes in dietary habits, and respondent characteristics. Results: A total of 25% of respondents agree they waste more food since beginning the medication, while 61% disagree. Respondents are significantly less likely to agree with this statement if they have been on the medication a longer time and are significantly more likely to agree if they reported experiencing nausea since beginning the medication. Dietary changes consistent with more vegetable intake are also significantly associated with less waste. Conclusions: Uptake of a novel class of anti-obesity medications may significantly affect food waste patterns. With the potential for widespread adoption of such medications, and given the societal import of reducing food waste, understanding the interaction of these two consumer trends is critical for projecting their joint impact on the food system and for equipping new GLP1A users to limit food waste.

## 1. Introduction

The use of anti-obesity medications (AOMs) is on the rise in the United States with the introduction of glucagon-like peptide-1 receptor agonists (GLP1As) to the pharmaceutical market. In clinical studies, GLP1As have been shown to impact patients’ eating habits by reducing appetite, slowing down gastric emptying, and shifting food preferences [1,2]. GLP1As have demonstrated a plethora of potential benefits, including treatment for diabetes, weight loss, and treatment for other obesity-related conditions [3]. With continued success in clinical trials, medications such as semaglutide, liraglutide, and tirzepatide (which also features glucose-dependent insulinotropic polypeptide, a second key ingredient) are quickly becoming popular treatments for weight loss [4]. GLP1A use has been increasing in prevalence, with about 12% of U.S. adults reporting having taken at least one dose of a GLP1A and 6% reporting active use, along with 59% of adults claiming to have heard “some” or “a lot” about these medicines [5]. Our study explores whether the uptake of this medication also has a significant relationship with food waste.

Food waste is a pressing global issue with significant environmental, economic, and social implications [6]. This waste not only represents a substantial inefficiency in the food supply chain but also contributes to greenhouse gas emissions and depletes natural resources [7]. In the United States, the average consumer wastes 32% of purchased food, valued at $240 million annually [8]. Various factors contribute to food waste, including consumer behavior, improper storage, and over-purchasing [9,10], which are each intricately intertwined with consumers’ dietary decisions. With the increasing prevalence of anti-obesity medications, which are known to alter eating habits by reducing appetite and changing food preferences [11], there is a potential intersection between AOMs and food waste.

The purpose of this study is to assess how consumers alter the rate of food waste after beginning GLP1As and to identify factors associated with this relationship. As individuals taking these medications may purchase and consume less food, and possibly change the types of food that they consume [1,2], understanding how these changes impact food waste is crucial. By examining this intersection, we hope to provide insights that could prepare new AOM patients with strategies for reducing food waste and help predict aggregate food waste trends as AOMs become more widely prescribed.

## 2. Materials and Methods

We surveyed 505 U.S. adults who reported current use of a GLP1A. The self-administered online questionnaire focused on sociodemographic factors; personal characteristics; and questions inquiring about changes in dietary habits, weight, and food waste since beginning the AOM. The questionnaire was carefully reviewed by the investigators prior to use but has not been assessed for reliability due to the novelty of the topic. Human subjects’ approval was granted by the Ohio State University Internal Review Board (2024E0009), and all participants provided informed consent. Data were collected from respondents from throughout the United States during April 2024 via Qualtrics software (Qualtrics, Provo, UT, USA) with participants recruited via the Prolific recruitment platform (www.prolific.com, April 2024). The questionnaire is available in the Supplementary Materials as are the data used in the current analyses.

The focal (dependent) variable is a respondent’s answer to the question, “Indicate your level of agreement with the following statement: Since beginning this medication I have found I waste more of the food that I purchase”. Responses were indicated on a 5-point scale where strongly disagree was coded −2, somewhat disagree as −1, neither agree nor disagree as 0, somewhat agree as 1, and strongly agree as 2.

One key set of explanatory variables includes whether respondents indicated suffering from a list of known side effects associated with GLP1As since beginning the medication, including nausea, vomiting, diarrhea, abdominal pain, constipation, injection site redness, low blood sugar, or other side effects. Indicated side effects were coded as 1 and all others as 0. Another set of explanatory variables includes responses to a question about changes in consumption of particular food categories since beginning the medication, including carbohydrates, proteins, alcohol, healthy fats, fried foods, savory foods, sweet foods, fruits, vegetables, fish, meats, dairy, and pasta and rice. Participants could indicate that, since beginning the medication, they consumed more (coded 1), about the same amount (coded 0), or less (coded −1) of each food category. Another set of explanatory variables includes whether respondents started adherence to a particular dietary regimen since beginning the medication, with the options being paleo, Atkins, Dukan, intermittent fasting, vegetarian, vegan, pescatarian, Mediterranean, Kosher, halal, low-fat, or other, with those who started such a diet since beginning the AOM coded as 1 and 0 otherwise.

Additional control variables include the type of medication (semaglutide, liraglutide, tirzepatide, or other/unsure); the length of time on the medication (<90 days, 90–180 days, 181–365 days, or >365 days); and the respondent’s sex, age, education, race, ethnicity, employment status, and medical insurance status. Respondent weight change since beginning the medication was strongly correlated with respondent length of time on the medication, and hence was omitted to avoid multi-collinearity in the regression analysis and because weight change was not reported by all respondents.

The relationship between the dependent variable and the explanatory variables was assessed via a multivariate regression model estimated via ordinary least squares (OLS), where inferences concerning statistical significance are based upon robust standard errors. Given the inherently ordinal nature of the dependent variable, we also estimated an ordinal logit model, which leads to qualitatively similar inferences. For brevity and ease of interpretation, we present only the OLS results. All statistical work was conducted using Stata (version 18.0). Statistical significance was set at the 0.05 level.

## 3. Results

### 3.1. Sample Characteristics

The sample of 505 U.S. consumers includes substantial demographic and economic diversity (Table 1). In terms of racial composition, 63.8% identify as white/Caucasian, 25.3% identify as Black or African American, and 10.9% provide a different racial identification; in terms of ethnic identification, 9.3% identify as Hispanic or Latina/o. About half identify as male, with about one-third reporting age less than 35. In terms of economics, about 20% report annual household incomes below $50,000, while 76.8% report full-time employment, and 89.1% report having medical insurance. About 73% report having a bachelor’s degree or beyond.

Sample respondents report substantial dietary variation since beginning their AOM. Food categories that were eaten less since the onset of medication use include carbohydrates, pasta, alcohol, fried foods, savory foods, sweets, and dairy, while there was little movement in terms of meat consumption. Increased consumption was reported for all proteins, fish, healthy fats, fruit, and vegetables. The most popular diets started since beginning AOMs included low fat and intermittent fasting.

In terms of medication-related variables, about two-thirds of the respondents report semaglutide as their medication, with more than a quarter reporting they have been on the medication for more than a year. The average out-of-pocket cost of the medication reported is $175.40/month (90% confidence interval is $0–$800). The most frequently reported side effects from the medication include nausea (49%), constipation (32%), abdominal pain (29%), and diarrhea (25%).

### 3.2. Changes in Food Waste after GLP1A Uptake

About one-quarter of respondents agree with the statement that they waste more of the food they purchase since beginning their AOM, while about 61% disagree. The regression analysis (Table 2) reveals that respondents with nausea are significantly more likely to report agreement, while those who have been taking the medication for more than a year are significantly less likely to voice agreement than those who began the medication less than 90 days ago. Respondents who report increasing their vegetable intake since beginning the medication are significantly less likely to report an increase in food waste. The only respondent demographic characteristic that is significantly associated with the food waste variable pertains to education level, with those who have earned a bachelor’s degree having a significant negative relationship with the food waste variable compared with those with more or less formal education. No other demographic or economic variables are significantly related to the food waste variable.

## 4. Discussion

The findings provide insight into the relationship between self-assessed changes in food waste since a respondent began GLP1A use and plausible drivers of this relationship. Notably, individuals who experienced nausea were significantly more likely to report an increase in food waste. This association suggests that adverse medication effects can have an impact on food consumption and waste patterns. Nausea should lead to a reduction in appetite and, hence, an increased likelihood of having food that is no longer desired and thus discarded, which contributes to food waste.

**Figure 1:**
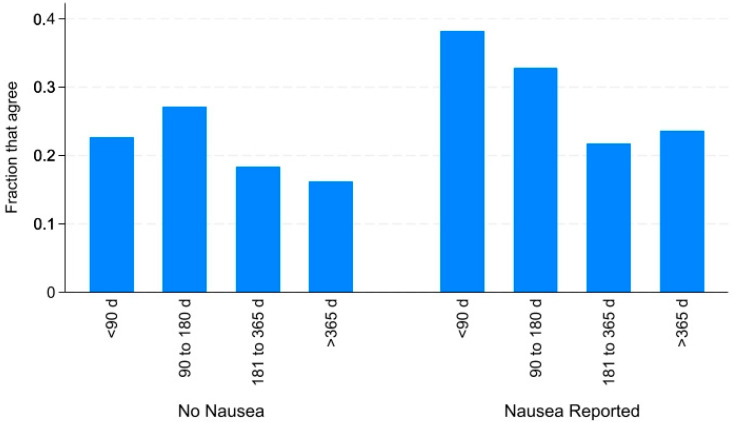
Fraction of sample that agrees that they waste more food since beginning medication by days on the medication and by the presence of nausea as a side effect of the medicine (N = 505).

The number of days on medication is also significantly related to perceived changes in food waste: those with a longer duration of use report less agreement that food waste has increased since commencing medication. This pattern is consistent with an acclimation of food purchasing and preparation behaviors in light of new dietary demands and preferences. Figure 1 displays the fraction of the sample that agrees with the statement concerning increased food waste since medication onset by the duration of their experience with the medication and by the presence or absence of nausea as a reported side effect. Whether the respondent reported the side effect of nausea or not, those who have been on their AOM for more than half a year report less agreement, though the magnitude of the effect appears larger among those who also report nausea from taking the medication.

One conjecture is that more food is wasted during initial exposure to the medication due to changes in dietary preferences and habits. Indeed, we find in Table 1 than the average diet of respondents changed to include more produce, protein, fish, and healthy fats and less alcohol, carbohydrates (including pasta), sweets, and dairy products. In particular, Table 2 reveals that individuals who reported consuming more vegetables as part of their post-medication dietary transition were significantly less likely to agree that they increased food waste since beginning a GLP1A. This suggests a potential that increased vegetable consumption may help combat food waste for those beginning GLP1As. Vegetables are among the most wasted of all food categories in the United States [12]. Transitioning to a more vegetable-intense diet may increase the likelihood of incorporating these items into meals rather than discarding them. This observation is consistent with the previous literature showing that healthy lifestyle changes—namely, preparation of food inside the household—contributes to reducing food waste [13].

We note that the regression coefficients for the remaining independent variables were not statistically different from zero. This includes the respondent’s type of medication (e.g., semaglutide vs. liraglutide) and medication side effects other than nausea. To the best of the authors’ knowledge, there is no extant literature assessing how different forms of GLP1As or medicine side effects relate to household food waste. No significant relationships were observed concerning post-medicine dietary changes. Previous studies have predicted positive associations between healthier diets and food waste in a cross-sectional simulation [14] as well as negative associations, with the negative association predicted to occur during post-COVID-19 activity restrictions when households had additional time to dedicate to food preparation [15].

There were no significant regression coefficients among variables related to respondent age, race, ethnicity, sex, employment status, insurance status, or household income. The only personal characteristic with a statistically significant association was that of education, where those reporting a bachelor’s degree have a significant negative relationship with the dependent variable when compared with respondents with less formal education. As this is the first exploration of how self-reported food waste changes after uptake of a GLP1A, there is no direct basis of comparison in the received literature. However, past studies have revealed significant relationships between the level of household food waste at a particular point in time and many of these factors. This literature reveals considerable variation across studies (see [9,16] for systematic reviews). For example, a recent systematic review [16] concludes that the literature yields no shared consensus about the relationship between household food waste and age, gender, income, or education, and notes that different studies have found positive, negative, or no significant relationships for many of these factors.

We note that there are several confounding factors that are not explored in this study. For example, we do not control for the residence of the survey participant. Previous studies have noted the role of urbanicity on food waste generation [17] as well as state-level differences across the United States [18]. Hence, the region of the country and the level of urbanicity are plausible drivers of changes in recent food waste. Also, because all data were collected at a single point in time, we are unable to control for the season of the response, which has previously been identified as a factor in household food waste levels [19] and, therefore, could plausibly influence recent changes in food waste.

## 5. Conclusions

Reducing food waste [6] and carrying less weight [20] are both supportive of improved sustainability. It would appear that there is an additional sustainability-supporting interaction between these trends in that only about a quarter of U.S. consumers report increasing food waste after taking anti-obesity medications, and this is further mitigated as patients are able to treat typical symptoms (nausea) and as they acclimate to the medication and the concomitant dietary changes over time. Hence, making new AOM patients aware that the uptake of the medication can lead to some additional waste, particularly if they suffer from nausea, may help them reduce expenditures on foods they will be unlikely to consume. Further, as more people turn to GLP1As over the coming years, we might look for impacts on the amount of food waste emerging from households so that models of food waste reduction goal attainment might be better calibrated.

This study is limited in several dimensions that should be addressed in subsequent research. First, the absolute amount of food that is wasted by respondent households was not gathered, which limits our ability to project how uptake of GLP1As will affect, e.g., municipal organic waste levels. Greater granularity should be obtained concerning the amounts and types of food that are wasted as patients progress through AOM treatment by using previously validated and reliable measures of food waste [12] and GLP1A adherence [21]. Second, a prospective study design featuring an appropriately powered sample that assesses behavior prior to and then at different stages of medication use would provide increased confidence in these results. Such a survey should also include potentially confounding variables omitted from this study such as respondent residence and should be collected during various seasons. Third, future studies should use designs that test messaging or other interventions designed to mitigate food waste among AOM patients. Such efforts would provide insights into approaches to enhance sustainability among this growing segment of the population.

## Figures and Tables

**Table 1:** Summary statistics.

Variable	Mean or %
More waste since beginning medication	
Strongly disagree	26.7
Somewhat disagree	34.1
Neither agree nor disagree	13.9
Somewhat agree	18.6
Strongly agree	6.7
Side effects	
Abdominal pain	28.7
Constipation	32.3
Low blood sugar	10.5
Nausea	49.3
Diarrhea	25.1
Injection site redness	15.0
Vomiting	16.0
Other	11.7
Change in consumption since beginning AOM *	
Carbohydrates	−0.49
Protein	0.49
Alcohol	−0.63
Healthy fats	0.25
Fried foods	−0.64
Savory foods	−0.17
Sweets	−0.49
Fruit	0.43
Vegetables	0.55
Dairy	−0.22
Fish	0.17
Meat	0.00
Pasta and rice	−0.41
Started diet since beginning medication	
Paleo	0.10
Atkins	0.11
Dukan	0.07
Intermittent fasting	0.25
Vegetarian	0.12
Vegan	0.11
Pescatarian	0.11
Mediterranean	0.12
Kosher	0.08
Halal	0.07
Low fat	0.26
Other	0.07
Type of medication	
Liraglutide	4.1
Semaglutide	68.5
Tirzepatide	22.4
Other/not sure	5.0
Out of pocket medication cost ($/month)	175.40
Days since beginning medication	
<90	29.9
90–180	22.6
181–365	21.0
365+	26.5
Age	
18–24	5.8
25–34	25.6
35–44	26.8
45–54	28.8
55–64	9.2
65+	3.8
Household income (annual)	
<$50,000	20.4
$50,000–$99,999	35.4
$100,000+	44.2
Education	
Less than bachelor’s degree	26.5
Bachelor’s degree	45.5
Beyond bachelor’s degree	27.9
Racial self-identification	
White/Caucasian	63.8
Black/African American	25.3
All other identifications	10.9
Employed full time	76.8
Has medical insurance	89.1
Identify as male	43.4
Identify as Hispanic or Latina/o	9.3

* Coded as “more since beginning medication” = 1, “about the same” = 0, and “less since beginning medication” = −1. The sample consists of 505 respondents, though only 503 reported age.

**Table 2:** Regression model: change in food waste since beginning medication (N = 503).

Variable	Coefficient	Robust Standard Error	*p*-Value
Side effects			
Abdominal pain	0.20	0.14	0.15
Constipation	−0.05	0.12	0.68
Low blood sugar	0.24	0.19	0.21
Nausea	0.26	0.12	0.02 *
Diarrhea	0.04	0.14	0.78
Injection site redness	−0.05	0.15	0.76
Vomiting	0.19	0.18	0.30
Other	0.15	0.19	0.43
Food consumption since beginning AOM			
Carbohydrates	0.06	0.11	0.62
Protein	0.06	0.11	0.60
Alcohol	0.18	0.12	0.12
Healthy fats	−0.12	0.11	0.26
Fried foods	0.16	0.13	0.22
Savory foods	−0.10	0.10	0.32
Sweets	0.14	0.12	0.25
Fruit	−0.05	0.11	0.63
Vegetables	−0.27	0.13	0.04 *
Dairy	0.03	0.10	0.73
Fish	−0.05	0.11	0.63
Meat	0.07	0.10	0.49
Pasta	−0.14	0.11	0.23
Started diet since beginning medication			
Paleo	0.04	0.37	0.91
Atkins	0.12	0.22	0.59
Dukan	0.42	0.28	0.14
Intermittent fasting	−0.13	0.15	0.40
Vegetarian	0.22	0.23	0.34
Vegan	−0.46	0.24	0.06
Pescatarian	−0.20	0.23	0.37
Mediterranean	0.07	0.18	0.69
Kosher	0.01	0.26	0.98
Halal	−0.00	0.32	0.99
Low fat	0.10	0.15	0.49
Other	−0.02	0.26	0.95
Type of medication			
Liraglutide (omitted category)	-	-	-
Semaglutide	0.10	0.31	0.74
Tirzepatide	0.21	0.33	0.52
Other/not sure	0.24	0.38	0.54
Days since beginning medication			
<90 (omitted category)	-	-	-
90–180	−0.13	0.16	0.41
181–365	−0.22	0.16	0.16
365+	−0.35	0.15	0.03 *
Age			
18–24 (omitted category)	-	-	-
25–34	0.19	0.28	0.49
35–44	−0.00	0.29	0.99
45–54	0.15	0.29	0.62
55–64	−0.17	0.33	0.61
65+	0.06	0.39	0.89
Household income (annual)			
<$50,000 (omitted category)	-	-	-
$50,000–$99,999	0.15	0.16	0.35
$100,000+	0.07	0.16	0.64
Education			
Less than bachelor’s degree (omitted)	-	-	-
Bachelor’s degree	−0.38	0.15	0.02 *
Beyond bachelor’s degree	0.20	0.18	0.26
Racial self-identification			
White/Caucasian	0.08	0.19	0.66
Black/African American	0.30	0.23	0.19
All other identifications (omitted)	-	-	-
Employed full time	−0.00	0.15	0.98
Has medical insurance	−0.05	0.18	0.79
Identify as male	0.07	0.13	0.59
Identify as Hispanic or Latina/o	−0.24	0.18	0.19
R^2^	0.15		

* Denotes statistically significant coefficients.

## Data Availability

The data and statistical code used in this study are available in the Supplementary Materials.

## Supplementary Materials

The following supporting information can be downloaded at: https://www.mdpi.com/article/10.3390/nu16193274/s1, Consumer Survey, Data, Statistical code.
