# Supplementary material for: Changes in Food Waste among a Sample of U.S. Consumers after Beginning Anti-Obesity Medication
Source: Nutrients. 2024 Sep 27;16(19):3274. doi: 10.3390/nu16193274 (PMC11478882; doi:10.3390/nu16193274)
Supplement: Supplementary file 1 [file nutrients-16-03274-s001.zip › GLP_Food_Demand_Survey (1).pdf]

GLP Food Demand Survey

Start of Block: consent

consent Study Title: Weight Loss Food Demand Study
Principle Investigator: Brian E. Roe
Study Sponsor: Ohio State University

Researchers from The Ohio State University would like to ask you some questions about your experiences and results before and after beginning a medication known as Glucagon-like peptide-1 (GLP-1) receptor agonist, including questions about your food purchases, food consumption, and food waste and questions about the effects of medication on your health and wellness. These questions are being asked by Professor Brian Roe of Ohio State University and will take about 5 - 10 minutes to answer.

The survey requires you to complete both multiple choice and open-ended (write-in) questions. Open-ended questions should require brief responses, but the length of the response is up to you. This study does not require the study coordinator to access any of your personal information. You will not be asked any information that can identify who you are. Your survey responses, but no information that can identify who you are, may be shared with other researchers in the future. Information provided to this study does not have the potential to damage your financial standing, employability or reputation, or place you at risk of criminal or civil liability.

We will work to make sure that no one sees your survey responses without approval. But, because we are using the Internet, there is a chance that someone could access your online responses without permission. In some cases, this information could be used to identify you.

Your participation is voluntary, and you may skip any question or request for any reason. Refusal to participate will involve no penalty or loss of benefits to which you are otherwise entitled. You may choose to stop participating at any time without penalty or loss of benefits to which you are otherwise entitled. There are no direct benefits to you from participating. You will receive compensation at the level highlighted when you clicked upon the offer to take the survey.

If you have questions about the research, or feel you have been harmed by study participation, you may contact Brian Roe at roe.30@osu.edu. For questions about your rights as a participant in this study or to discuss other study-related concerns or complaints with someone who is not part of the research team, you may contact the Office of Responsible Research Practices at hsconcerns@osu.edu.

Do you consent to participate?

○ Yes (1)

○ No (2)

End of Block: consent

Start of Block: ID

id What is your Prolific ID? Please note that this response should auto-fill with the correct ID.

____

End of Block: ID

Start of Block: Survey

Q1 What category of weight loss medication are you currently taking? (select one)

○ Semaglutide (e.g., Ozempic, Wegovy, Rybelsus) (1)

○ Tirzepatide (e.g., Mounjaro or others) (2)

○ Liraglutide (e.g., Saxenda or others) (3)

○ Not sure (4)

Page Break

JS

Q2 When did you first start taking this medication?

____

Page Break

*

Q3 About how much do you spend (out of pocket) each month to acquire this medication?

	$/month (1)
Please enter: (1)	

Page Break

Q4 What type of side effects have you experienced since starting the medication? (select all that apply)

- [ ] Nausea (1)
- [ ] Vomiting (2)
- [ ] Diarrhea (3)
- [ ] Abdominal pain (4)
- [ ] Constipation (5)
- [ ] Injection site redness (6)
- [ ] Low blood sugar (7)
- [ ] Other side effects (8)
- [ ] ⊗None (9)

Page Break

*

Q5 How much did you weigh when you started taking the medication?

	Pounds (1)
Please enter: (1)	

*

Q6 What is your current weight?

	Pounds (1)
Please enter: (1)	

*

Q7 What is your current height?

	Feet (1)	Inches (2)
Please enter: (1)		

Page Break

Q8 Compared to the time before beginning the medication, have you changed the average number of calories you consume each day?

- Yes (1)
- Unsure (2)
- No (3)

Page Break

Display This Question:

If Compared to the time before beginning the medication, have you changed the average number of calo... != No

*

Q9 What was/is your average daily calorie consumption?

	Calories / day (4)
Before beginning the medication (3)	
Currently (6)	

Page Break

Display This Question:

If Compared to the time before beginning the medication, have you changed the average number of calo... = No

*

Current_Calories What is your current average **daily** calorie consumption?

____

Page Break

Q10 Has the nutritional content or profile of your typical daily food and drink intake changed compared to the time before you began the medication?

- Yes (1)
- Unsure (2)
- No (3)

Page Break

Display This Question:

If Has the nutritional content or profile of your typical daily food and drink intake changed compar... = Yes

Q11 Would you say that you daily intake of food and drink has become (select one):

- ◯ Much healthier/nutritious (1)
- ◯ Somewhat healthier/nutritious (2)
- ◯ Somewhat less healthy/nutritious (3)
- ◯ Much less healthy/nutritious (4)

Page Break

Q12 Consider the food categories below. Would you say that you consume more or less of each since you began the medication?

	More (1)	About the same (2)	Less (3)
Carbohydrates (1)	○	○	○
Proteins (2)	○	○	○
Alcohol (3)	○	○	○
Healthy fats (e.g. avocados) (4)	○	○	○
Fried foods (5)	○	○	○
Savory foods (6)	○	○	○
Sweet foods (7)	○	○	○
Fruits (8)	○	○	○
Vegetables (9)	○	○	○
Dairy (13)	○	○	○
Fish (10)	○	○	○
Meats (11)	○	○	○
Pasta and Rice (12)	○	○	○

Page Break

Q13 About how much money is usually spent by your household each week for food and drinks from places like restaurants, bars, and fast food places, including carry-out and delivery? Please include the cost of any alcoholic beverages.

	$/week (1)
Before beginning the medication: (1)	
Currently (2)	

Page Break

*

Q14 How much money is usually spent by your household each week for beer, wine, and other alcohol to be served at home?

	$/week (1)
Before beginning the medication: (1)	
Currently: (2)	

Page Break

Page Break

*

Q15 How much does your household usually spend each week for groceries, including food and non-food items?
Please include: In-person and online grocery shopping **and delivery service fees** Drinking water and delivery fees, if applicable Prepared meal kits Personal health and wellness items, diapers, pet food, and home cleaning supplies Grocery home delivery Purchases made with government benefits such as SNAP and WIC EBT cards, if applicable.
DO NOT include prescription drugs, alcohol, cigarettes, or other tobacco products.

	$/week (1)
Before beginning the medication: (1)	
Currently: (2)	

Page Break

Q16 Indicate your level of agreement with the following statements. Since beginning this medication I have found:

	Strongly agree (1)	Somewhat agree (2)	Neither agree nor disagree (3)	Somewhat disagree (4)	Strongly disagree (5)
The types of food I eat have changed (1)	o	o	o	o	o
I select smaller portion sizes for myself during meals (3)	o	o	o	o	o
I eat fewer times each day (4)	o	o	o	o	o
I buy food that is more expensive on a per pound basis (5)	o	o	o	o	o
I waste more of the food that I purchase (6)	o	o	o	o	o
I buy a smaller quantity of food (7)	o	o	o	o	o
I spend less money to buy the food I need (8)	o	o	o	o	o
I spend more money at the pharmacy to help me treat the side effects of this medication (9)	o	o	o	o	o

My budget has gotten tighter because of the expense of this medication (10)	○	○	○	○	○

Page Break

Q17 When considering the cost of this medication, the change in the costs of food, the change in the costs of any pharmacy expenditures to deal with side effects, and any other changes in your regular costs related to taking this medication, how much would you say that your monthly costs have changed since beginning this medication?

○ Increased by (1) ____

○ About unchanged (2)

○ Decreased by (3) ____

Page Break

JS

Q17 Which of the following diets or dietary patterns did you or do you follow (select one per row)?

	Before beginning this medication (1)	Since beginning this medication (2)	Neither (3)
Paleo (1)	☐	☐	☐
Atkins (2)	☐	☐	☐
Dukan (3)	☐	☐	☐
Intermittent fasting (4)	☐	☐	☐
Vegetarian (5)	☐	☐	☐
Vegan (6)	☐	☐	☐
Pescatarian (7)	☐	☐	☐
Mediterranean (8)	☐	☐	☐
Kosher (9)	☐	☐	☐
Halal (10)	☐	☐	☐
Low fat (12)	☐	☐	☐
Other (11)	☐	☐	☐

Display This Question:

If Which of the following diets or dietary patterns did you or do you follow (select one per row)? = Other [Before beginning this medication]

Or Which of the following diets or dietary patterns did you or do you follow (select one per row)? = Other [Since beginning this medication]

Q18 You selected that you followed/follow 'Other' diet or dietary pattern , please describe the diet or dietary pattern you followed/follow in more detail below:

____

End of Block: Survey

Start of Block: Demographics module

age Which of the following categories best describes your age? Select one.

- ◯ 18-24 (1)
- ◯ 25-34 (2)
- ◯ 35-44 (3)
- ◯ 45-54 (4)
- ◯ 55-64 (5)
- ◯ 65 and older (6)

Page Break

sex What was your sex assigned at birth?

- ○ Male (1)
- ○ Female (2)

Page Break

gender How do you currently describe yourself?

- ○ Man (1)
- ○ Woman (2)
- ○ Non-binary (3)
- ○ Transgender man/Female-to-male (FTM) (4)
- ○ Transgender woman/Male-to-female (MTF) (5)
- ○ Gender non-binary/Genderqueer/Gender nonconforming (6)
- ○ Agender (7)
- ○ Bigender (8)
- ○ Prefer not to answer (9)

Page Break

Hisp_Lat_Span Are you of Hispanic, Latino, or Spanish origin?

- ○ Not of Hispanic, Latino/a, or Spanish origin (1)
- ○ Hispanic Mexican, Mexican American, or Chicano/a (2)
- ○ Hispanic Puerto Rican (3)
- ○ Hispanic Cuban (4)
- ○ Other Hispanic, Latino/a, or Spanish Origin (5)

Page Break

Race Which race would you consider yourself? (select all that apply)

- ○ White (1)
- ○ Black or African American (2)
- ○ American Indian or Alaska Native (3)
- ○ Asian - Asian Indian (4)
- ○ Asian - Chinese (5)
- ○ Asian - Filipino (6)
- ○ Asian - Japanese (7)
- ○ Asian - Korean (8)
- ○ Asian - Vietnamese (9)
- ○ Asian - Other (10)
- ○ Pacific Islander - Native Hawaiian (11)
- ○ Pacific Islander - Guamanian or Chamorro (12)
- ○ Pacific Islander - Samoan (13)
- ○ Pacific Islander - Other (14)
- ○ Other (15)
- ○ Don't know (16)

Page Break

Display This Question:

If Which race would you consider yourself? (select all that apply) = Other

Race_other Please enter other race:

____

Page Break

*

hhsize Including yourself, how many people live in your household?

____

Page Break

*

hhsize_decompose Among the ${hhsize/ChoiceTextEntryValue} people live in your household, please indicate the number of people in each of the following categories.

Children 0-5 years of age: : ________ (1)

Children 6-17 years of age: : ________ (2)

Male adults 18 years or older: : ________ (3)

Female adults 18 years or older: : ________ (4)

Total : ________

Page Break

Highest_educ What is the highest grade or level of school you have completed? Select one.

- ○ Less than 12th grade, NO DIPLOMA (1)
- ○ High school graduate, DIPLOMA or GED (2)
- ○ Some college or Associate degree (3)
- ○ Bachelor’s degree (4)
- ○ Graduate or professional degree (5)

Page Break

employment_status How would you best describe your employment situation? Select one.

- ○ Full-time employment (35 hours a week or more) (1)
- ○ Part-time employment (less than 35 hours a week) (2)
- ○ Unemployed (3)
- ○ Student (4)
- ○ Retired (5)
- ○ Unable to Work (6)

Page Break

Display This Question:

If How would you best describe your employment situation? Select one. = Full-time employment (35 hours a week or more)

Or How would you best describe your employment situation? Select one. = Part-time employment (less than 35 hours a week)

work_location Do you work from home, or travel to an office or other location(s) outside the home?

- ◯ Work from home (1)
- ◯ Travel to an office or other location(s) (2)
- ◯ Both work from home and travel to office or other location(s) outside the home (3)

Page Break

hh_income Which of the following categories best describes your annual household income before taxes for the previous tax year? Select one.

- Less than $10,000 (1)
- $10,000 - $19,999 (2)
- $20,000 - $29,999 (3)
- $30,000 - $39,999 (4)
- $40,000 - $49,999 (5)
- $50,000 - $59,999 (6)
- $60,000 - $69,999 (7)
- $70,000 - $79,999 (8)
- $80,000 - $89,999 (9)
- $90,000 - $99,999 (10)
- $100,000 - $149,999 (11)
- More than $150,000 (12)

Page Break

health_insured Are you covered by health insurance obtained through employment or purchased directly or by government programs like Medicare and Medicaid that provide Medical care or help pay medical bills?

- ○ Yes (1)
- ○ Unsure (2)
- ○ No (3)

End of Block: Demographics module
